# Behcet's Disease With Cerebral Artery Infarction Caused by Cerebral Arteritis as an Early Symptom Only With Elevated Interleukin-8

**DOI:** 10.3389/fneur.2019.01102

**Published:** 2019-10-22

**Authors:** Hao Yin, Yun Song, Meimei Zheng, Ju Han, Jiyou Tang

**Affiliations:** ^1^Shandong Provincial Qianfoshan Hospital, Shandong University, Jinan, China; ^2^The First Affiliated Hospital of Shandong First Medical University, Jinan, China

**Keywords:** Behcet's disease, neuro-Behcet's disease, cerebral infarction, cerebral arteritis, IL-8

## Abstract

**Background:** Behcet's disease (BD) is multi-systemic vasculitis, which generally is repeated oral and genital ulcerations as well as ocular and skin lesions. Today, the pathogenesis of BD remains mostly unknown. It is also suggested that the disease is probably related to autoinflammatory and autoimmune disorders, and innate immunity damages were perceived as key in its pathologic process. Only 5% of BD patients have neurological involvement, and it usually occurs in 4–6 years after the initial symptoms. Early onset of neurological impairment makes it difficult to diagnose and treat definitely.

**Case Presentation:** A 38-year-old man was admitted to our hospital with numbness and weakness of the left extremities. Diffusion magnetic resonance imaging (MRI) revealed focal infarction in the posterior limb of the internal capsule. Skin pathology suggested small vessel vasculitis, and high-resolution MRI revealed intracranial arteritis. The patient had a negative skin pathery test and then developed a scar at the venous puncture site at the early stage of disease. Laboratory examination showed that interleukin 8 (IL-8) increased. The patient was treated with an immunosuppressive agent including mycophenolate mofetil, hydroxychloroquine, and colchicine. All symptoms were alleviated after half a year's treatment. There was neither stroke nor recurrence of oral ulcer thereafter.

**Conclusion:** This case demonstrates that neurological involvement might be an early symptom of BD. IL-8 could act as a novel target for the treatment of BD theoretically and probably play a key role in disease recovery.

## Introduction

Behcet's disease (BD) is recognized as a multi-systemic disease characterized by possible inflammatory lesions of any-size vessels (1). Although the etiopathogenesis has not yet been clarified, it is generally believed that immunological factors play an important role (2–4). It is also suggested that the disease is probably related to autoinflammatory and autoimmune disorders, and innate immunity damages were perceived as having a key role in its pathologic process (5–7). The diagnosis is established by clinical criteria only (8). The international criteria for BD (ICBD) allow for earlier recognition, thus leading to earlier and accurate diagnosis and treatment with salutary results (9).

Involvement of central nervous system symptoms is reported in 3.2–49%, mostly about 5%, in BD patients (10, 11), which is known as neuro-Behcet's disease (NBD).Nervous system impairment mostly occurs in 4–6 years after the onset of BD (12). However, some of the patients have neurological symptoms at the same time as or even before the classical BD symptoms, which may lead to diagnostic confusion and may result in there being more BD patients than actually reported (13).

In this kind of patient, the pathological feature is various types of arteriovenous vasculitis, which mainly affects small vessels in multiple systems. It appears as focal or multiple brain parenchymal damage in the central nervous system (14, 15). Artery involvement is rare compared with venous involvement, manifesting as arterial stenosis and aneurysm formation, which often lead to arterial infarction or intracranial and/or subarachnoid hemorrhage (16–18).

## Case Presentation

**Figure 1:**
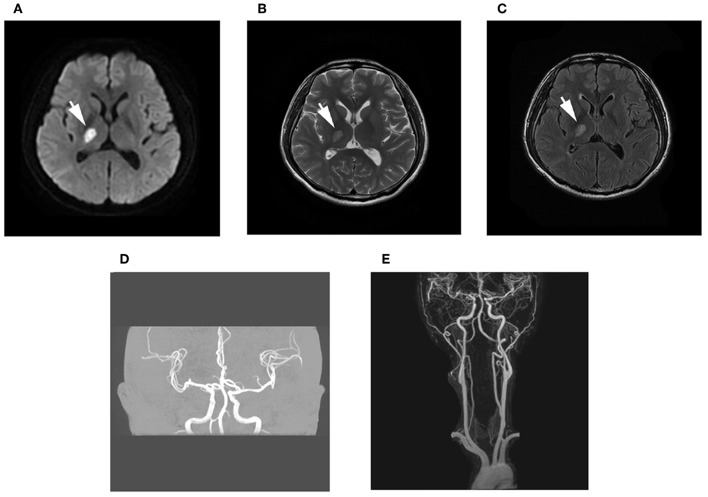
Brain magnetic resonance imaging (MRI). **(A–C)** Diffusion-weighted imaging (DWI) and T2 fluid-attenuated inversion recovery (FLAIR) imaging showed high signal, while long signal was shown in T1-weighted imaging and T2-weighted imaging of the posterior limb of the internal capsule. **(D,E)** Magnetic resonance angiography examination of the intracranial and cervical vascular was normal.

A 38-year-old man was admitted to our hospital with numbness and weakness of the left extremities. Diffusion-weighted imaging (DWI) revealed focal infarction in the posterior limb of the internal capsule, and cranial and cervical magnetic resonance angiography was normal (Figure 1).

**Figure 2:**
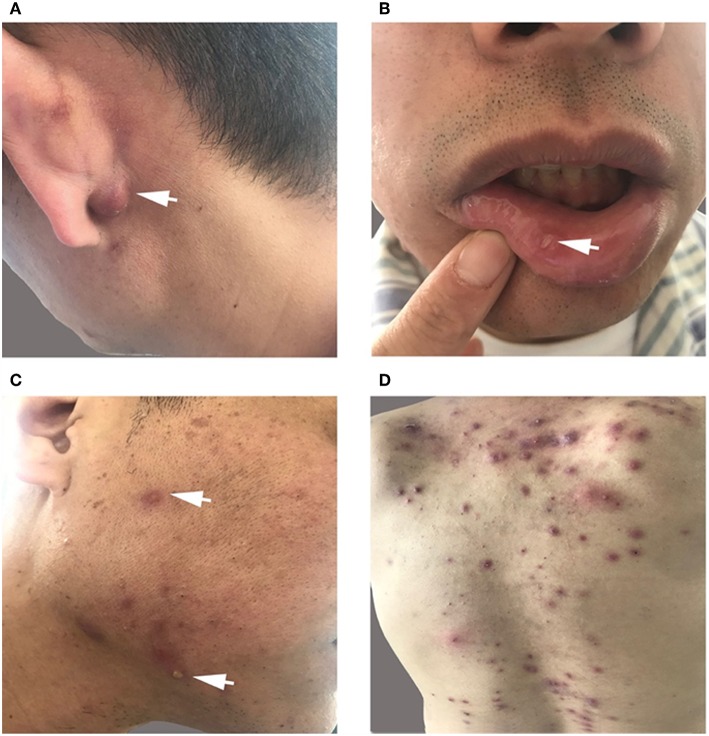
**(A,C,D)** Old and new acneiform folliculitis behind the ears **(A)** and on the face **(C)** and back **(D)**. **(B)** Recurrent multiple and painful oral ulcers.

**Figure 3:**
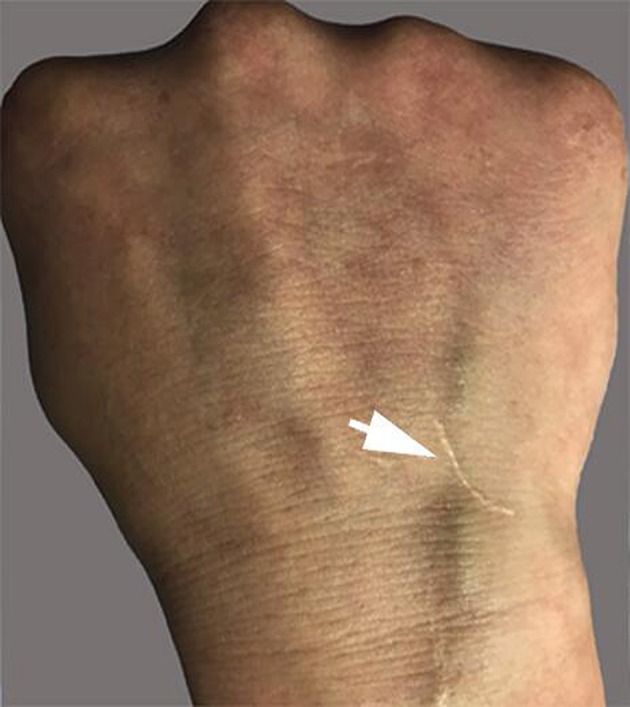
Scar at the venous puncture site.

The patient had recurrent multiple and painful oral ulcers after 2 months; meanwhile, he suffered pain in both knees and the left shoulder joint. The physical examination revealed that the old and new *acneiform folliculitis* were alternated on the face and back and behind the ears (Figure 2); also, a scar was shown at the venous puncture site (Figure 3). The patient had no other risk factors for cerebral atherosclerotic disease and is also not a smoker. Blood routine, serologic etiology test (including syphilis, AIDS, and other pathogens), lipid profile, plasma glucose, plasma and urine homocysteine levels (indicating the possibility of a genetic predisposition to thrombosis), proteins C and S deficiency, antithrombin III deficiency, activated protein C resistance, antiphospholipid antibody (hypercoagulability markers), anticardiolipin immunoglobulin G (IgG) and immunoglobulin M (IgM), antineutrophilic cytoplasmic antibody, C-reactive protein (CRP), erythrocyte sedimentation rate, and rheumatoid factor were performed, and all of them were within normal range. Transesophageal echocardiography, contrasted transthoracic echocardiography, and 24-h dynamic electrocardiogram were normal. Interleukin 8 (IL-8) was increased to 252 pg/ml (normal value: <62 pg/ml), but IL-6, tumor necrosis factors (TNF), and other cytokines were maintained at normal levels. No cardiogenic embolism, cardiac structure and rhythm abnormalities were found.

**Figure 4:**
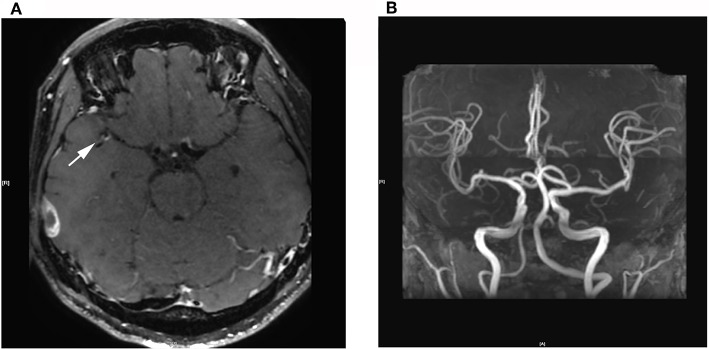
**(A)** 3D Cube T1 enhancement shows slightly thickened walls of the terminal portion of the M1 segment of the right middle cerebral artery. **(B)** 3D-TOF-MRA shows no abnormalities.

**Figure 5:**
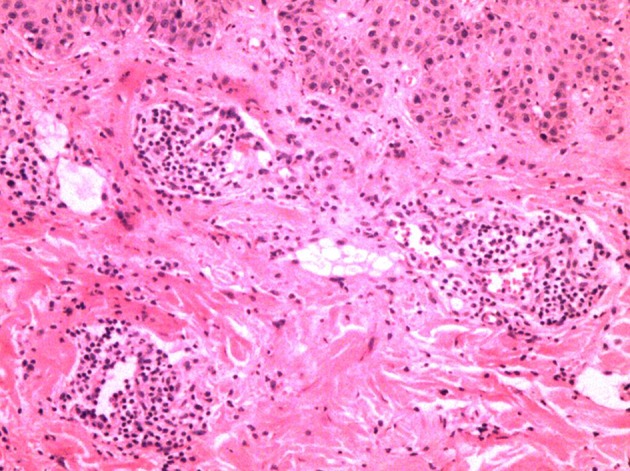
Extensively perivascular inflammatory cell infiltration with pathological biopsy of the skin lesion.

High-resolution magnetic resonance imaging (HRMRI) of the brain suggested that some blood vessel walls were slightly thickened, including the terminal portion of the left internal carotid artery, V4 segment of the bilateral vertebral artery, P2 segment of the right posterior cerebral artery (Figure 4), the terminal portion of M1 segment of the right middle cerebral artery (MCA), M2 and M3 segments of bilateral MCA, and A2 segment of the bilateral anterior cerebral artery. The enhancement scanning showed a double-track-like change and a narrowed vascular lumen, which suggested multiple intracranial arteritis. Hip magnetic resonance imaging (MRI) showed a long and flaky T1-weighted and T2 high fat-suppression signal on the lateral of the left femoral head, which suggested degenerative changes. Pathological biopsy of the skin lesion showed that some regions were infiltrated by extensively perivascular inflammatory cells, suggesting small vessel vasculitis (Figure 5). Genetic testing also was performed to analyze hereditary cerebrovascular disease elements (detected by Golden Field Medical, Inc., using analysis of missense mutations and alternative splicing; this lab has gotten the accreditation of the College of American Pathologists), and the result *did not* show pathogenic variation. Based on the above testing results and related to a history of genital ulcers of the patient, this case was diagnosed as BD under the direction of diagnostic criteria. The patient could not use azathioprine (AZP) to thiopurine S-methyltransferase (TPMT) mutation, and this could be checked by gene detection, which indicated that there was TPMT*3C (A719G) in this patient. Meanwhile, the patient refused to use cyclophosphamide after acquainting with its side effects (nephrotoxicity etc.) and its cumulative toxicity with long-term use (19).

Therefore, the patient was treated with mycophenolate mofetil dispersible tablets (Seccopin), hydroxychloroquine tablets (Flush), and colchicine tablets. After half a year's treatment, the patient's skin rash gradually was alleviated, and the symptoms of oral ulcer and nervous system damage did not recur.

## Discussion and Conclusions

Our patient fulfilled the diagnostic criteria of ICBD; he had recurrent oral ulcers (2 points), skin lesions (1 point), central nervous system lesions (1 point), vascular symptoms (1 point), and a positive pathergy test (1 point).

The patient initially consulted for acute cerebral infarction. This patient did not show risk factors for cerebral atherosclerotic disease other than smoking and also had no abnormalities in the gene test of hereditary cerebrovascular disease and other common causes, for example, hypercoagulability. According to his medical history, laboratory tests, and examination results, vasculitis caused by syphilitic vasculitis, rheumatoid arthritis, systemic lupus erythematosus, and Sjogren's syndrome can be excluded. The high-resolution MRI (HRMRI) of the brain showed intimal thickening in the wall of the intracranial and cervical arteries, which suggested arteritis. However, carotid dissection or Moyamoya symptoms *were not* shown. The new internal capsule lesion seen on MRI was consistent with the occlusion regions resulting from arteritis of the MCA deep perforator branches. In conclusion, the patient was diagnosed with BD.

Vasculitis is the central pathological process of BD. In this case, skin pathology was a representation of vasculitis, and it was verified by HRMRI as cerebral arteritis. Venipuncture is an inflammatory lesion of the skin, and it is similar to spontaneous skin lesions seen in the disease. Our patient had a negative skin pathery test, which developed a scar at the venous puncture site for the intravenous fluid infusion at the early stage of disease, but we have not observed the whole process of scar formation. The investigation results revealed that the presence of a positive reaction was irrelevant to the clinical manifestations of the disease (20, 21). However, the pathery reaction (PR) was reported to be significantly associated with vascular involvement (22). Chang et al. described that the intravenous Pathergy test seemed to be superior to the skin Pathergy test on specificity, sensitivity, and positive rate. Pathergy test reactions usually occur during active periods of BD as well (23). Although no peripheral vascular pathological examination was performed, the case was tested by negative classic PR, and the results suggested that the scar formation at the venipuncture site was caused by peripheral venous vasculitis.

Different immunologic abnormalities in the etiopathogenesis of BD have been reported. Increased Th1-associated cytokines such as IL-12, interferon-γ (IFN-γ), and tumor necrosis factor-α (TNF-α), as well as IL-2, IL-8, and IL-6, have been documented in BD patients (24). Many studies have reported that increased serum IL-8 plays an important role in BD (25–31). One recent report even implied that serum IL-8 could be a more credible marker of disease activity than CRP or erythrocyte sedimentation rate (ESR) (32). Durmazlar et al. found that IL-8 is positively correlated with the activity of BD and also closely related with the BD activity index and numbers of disease clinical manifestations. IL-8, known as neutrophil activating factor, is substantially produced by granulocytes, T cells, macrophages, and endothelial cells. A small amount of IL-8 can also be induced in keratinocytes, fibroblasts, chondrocytes, and hepatocytes (31, 33, 34). It strongly attracts and activates leukocytes. It has been supposed to establish a prominent connection between immune system activation and endothelial alterations involved in the conversion of mononuclear to granulocytic infiltration and causes raised adhesion of peripheral blood leukocytes to endothelial cells in BD. It has been found that the significant increase of IL-8 is associated with different clinical manifestations in BD, such as skin lesions, oral lesions, and ocular manifestations in one study (31). Moreover, compared with the active patient group without vascular involvement, the levels of IL-8 in patients with vascular involvement were markedly increased. On the basis of the patients' pathology and imaging, active BD patients with vascular involvement were found to possess pretty enhanced IL-8 in serum levels, which supported that endothelial cells may be partly responsible for secretion of IL-8 in the active phase of BD (35), since an IL-8 increase of fourfold in the active phase may represent endothelial injury in any part of the body. HRMRI results suggested central nervous system vasculitis. It is considered to be largely associated with a high IL-8 level, resulting in vascular endothelial destruction.

BD *is not* generally received as a cerebral vasculitis introducer compared with other systemic diseases known to lead to vasculitis. Only 1–5% arterial involvement is seen throughout the course of BD. The pattern of cerebral infarction and cerebral vasculitis represents a specific and uncommon form of neuro-Behcet's manifestation, and only two cases have been reported so far (10, 36). In one case, neurological deficits were considered to be infarction in the deep perforating branch of MCA, but it did not show specific change in brain imaging. The other case was an autopsy study, and it showed clearly that the infarction area was consistent with the occlusion regions resulting from arteritis of the MCA branches (37). Vascular involvement is a fatal complication for BD patients, so early diagnosis is very important.

Fortunately, for this case, the above conditions could be excluded according to the result that there was extensive cerebral artery intimal thickening from HRMRI. So we could clearly diagnose this case as BD with cerebral arterial infarction caused by cerebral arteritis, which led to early nervous system symptoms. In addition, we found that plasma IL-8 level was significantly elevated at the early stage of this case, while ESR, CRP, and other indicators have no obvious abnormalities. IL-6 is a key cytokine involved in NBD, especially since this inflammatory marker has been found to be increased also in the cerebrospinal fluid of patients with BD. However, IL-6 was not changed in the patient's plasma, which suggested that IL-8 is a dominant factor in the BD process.

AZP is the preferred treatment for BD. It commonly causes myelosuppression, presenting as megaloblastic anemia, leukopenia, severe hair loss, macrocytosis, pancytopenia, and thrombocytopenia (38–40). Leukopenia and severe hair loss are strongly associated with mutations in TPMT and NUDT15, which are widespread in the Asian population (41). Gene detection indicated that there was TPMT*3C (A719G) in this patient, so we did not choose AZP to treat patients. Mycophenolate mofetil is used in organ transplantation, multiple sclerosis, Crohn's disease and other immune-related diseases. Borhani Haghighi and Safari divide the anti-NBD armamentarium into first-line, second-line, and experimental drugs. Mycophenolate mofetil is classified as a second-line drug (42). The International Neuro-Behcet's Advisory Group has developed a consensus on NBD, which refers to mycophenolate mofetil as an alternative drug for NBD (43). Colchicine is used for the treatment of gout (44), as well as specific inflammatory conditions such as familial Mediterranean fever (FMF) (45), recurrent pericarditis (46) and BD (47). It seems to inhibit neutrophil chemotaxis and reduce plasma levels of cytokine, thereby reducing the occurrence and the duration of mucocutaneous (oral ulcers, erythema nodosum, genital) and articular manifestations of the disease (47). Hydroxychloroquine is proven to have a relatively benign toxicity profile and a low incidence of infections as an immunosuppressive agent (48, 49). A large cohort study has reported the successful empiric use of hydroxychloroquine to treat BD (50).

Considering the choice of patients and the drugs, the inflammatory activity was controlled followed by treatment with mycophenolate mofetil dispersible tablets (Seccopin), hydroxychloroquine tablets (Flush), and colchicine tablets. The clinical symptoms were ameliorated, such as suppressing oral immunity, preventing relapses in skin lesions and progressive neurological disease, as well as settling the systemic disease. However, it is unclear whether long-term immunosuppression might also work well in central nervous system (CNS) vasculitis. That is to say, the risk of recurrent cerebral infarcts remains to be elucidated in the future. Therefore, seeking new medicine targets becomes an urgent issue. IL-8, a major chemoattractant and activator of neutrophils, could act as a novel target for the treatment of BD theoretically and perhaps prove to be the key to recovery.

## Data Availability Statement

Publicly available datasets were analyzed in this study. This data can be found here: hospital ID: 000444000, Shandong Povincial Qianfoshan Hospital.

## Ethics Statement

The studies involving human participants were reviewed and approved by the ethics committee of Qianfoshan Hospital. The patients/participants provided their written informed consent to participate in this study. Written informed consent was obtained from the individual(s) for the publication of any potentially identifiable images or data included in this article.

## Author Contributions

HY, YS, and MZ made substantial contributions to conception and design. HY and MZ collected the data. HY, YS, MZ, and JH contributed to analysis and interpretation of the data. HY, JH, and JT have been involved in drafting the manuscript or revising it critically for important intellectual content. All authors have given final approval of the version to be published. Each author should have participated sufficiently in the work to take public responsibility for appropriate portions of the content. All authors agreed to be accountable for all aspects of the work in ensuring that questions related to the accuracy or integrity of any part of the work are appropriately investigated and resolved.

### Conflict of Interest

The authors declare that the research was conducted in the absence of any commercial or financial relationships that could be construed as a potential conflict of interest.

## Abbreviations

IL-8: interleukin 8
NBD: neuro-Behcet's disease
BD: Behcet's disease
ICBD: international criteria for Behcet's disease
AZP: azathioprine
MMF: mycophenolate mofetil
IL-1: interleukin 1
IgG: immunoglobulin G
IgM: immunoglobulin M
DWI: diffusion-weighted imaging
MRI: magnetic resonance imaging
HRMRI: high-resolution MRI
MCA: middle cerebral artery
CRP: C-reactive protein
ESR: erythrocyte sedimentation rate
TPMT: thiopurine S-methyltransferase
FMF: familial Mediterranean fever.
